# Do China’s Urban–Environmental Quality and Economic Growth Conform to the Environmental Kuznets Curve?

**DOI:** 10.3390/ijerph182413420

**Published:** 2021-12-20

**Authors:** Wenhao Song, Chunhui Ye, Yuheng Liu, Weisong Cheng

**Affiliations:** 1 China Academy for Rural Development, Zhejiang University, Hangzhou 310058, China; songwenhao@zju.edu.cn (W.S.); chws@zju.edu.cn (W.C.); 2 School of Public Affairs, Zhejiang University, Hangzhou 310058, China; 3 School of Economics and Finance, Xi’an Jiaotong University, Xi’an 710061, China; lyhtsune@163.com

**Keywords:** urban–environmental quality, economic growth, EKC, environmental pollution index, environmental absorption index

## Abstract

The environmental Kuznets curve (EKC) expresses the relationship between environmental quality and economic growth. Based on the defects of previous studies on EKC using only environmental pollution indicators, this study holds that environmental quality is the result of pollutants after treatment, absorption, and self-purification, including two dimensions of pollution and absorption. Therefore, the environmental pollution and absorption data of 74 key environmental monitoring cities in China from 2005 to 2017 were selected, and a comprehensive index of environmental quality was constructed using the vertical and horizontal grading method. Then, based on the relevant economic growth indicators of these cities, they were divided into first-tier and new first-tier, second-tier, third-tier, and below. It was found that the EKC of the total sample, the first-tier and new first-tier cities, and the second-tier cities presented an inverted N-shape and had passed the second inflection point, where environmental quality continues to improve with the deepening of economic growth. There was no EKC in the third-tier and below cities. The findings have important implications. China can cross the second inflection point of the EKC and improve environmental quality at a low income level. Therefore, by vigorously developing cleaner production technologies and raising residents’ awareness of environmental protection, it is possible to improve environmental quality at a lower income level than expected, which provides a useful reference for other developing countries.

## 1. Introduction

Environmental quality is inseparable from economic growth [1]. On the one hand, due to the exploitation of natural resources and the increase in pollutant emissions, economic growth will lead to the decline of environmental quality [2]; on the other hand, the negative externalities of environmental quality deterioration also limit long-term sustainable economic growth [3]. Therefore, balancing the relationship between economic growth and environmental quality to maintain high-quality economic growth and stabilize environmental quality has become an important issue in the development path of various countries.

With global climate change, China has encountered environmental problems frequently over recent years, delivering great impact on production and life. In particular, land desertification, winter haze, and sharp declines in biodiversity have obviously affected the normal development order. Western developed countries generally adopted the model of “grow first and clean up later” in their development processes, thus having paid a heavy price [4]. Therefore, China has to take these lessons and search for a “win–win” path for economic growth and environmental quality [5]. Since the 18th CPC National Congress, more attention has been paid to environmental quality, while constructing ecological civilization has been promoted to a national strategy. President Xi Jinping emphasized that “Clear waters and green mountains are as good as mountains of gold and silver”. In addition, the parallel development mode for both economic development and environmental protection is also urgently required to be integrated with all aspects of industries’ production and people’s daily life. Therefore, exploration of the context of environmental quality and economic growth will undoubtedly create profoundly theoretical and practical significance for high-quality economic development in the future. This study is purposed to verify the existence of urban environmental Kuznets curves in China. On the one hand, it will enrich the relevant theoretical results of environmental Kuznets curves; on the other hand, it will provide policy enlightenments for other developing countries.

**Figure 1:**
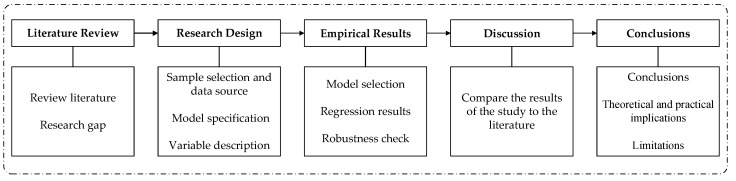
Research roadmap.

The remainder of the paper is organized as follows: Section 2 presents a literature review about EKC. The research design part in Section 3 explains the sample selection, data source, model setting, and variables. Section 4 presents the results of the empirical estimation. Section 5 presents a discussion of the main findings. The conclusions are then presented in Section 6. The research roadmap of this study is shown in Figure 1.

## 2. Literature Review

### 2.1. EKC on Different Scales

Since the 1990s, many studies have explored the relationship between economic growth and environmental quality. The most important discovery in empirical research is the environmental Kuznets curve (EKC); it is an inverted U-shaped relationship between economic growth and environmental pollution [6]. Environmental quality will deteriorate in the early stages of economic growth and improve in the later stages of economic growth. This relationship has also been confirmed in the development of many developed and industrialized countries [7,8,9,10,11,12]. For example, Destek and Sarkodie [13] examined the validity of the EKC hypothesis by investigating the relationship between economic growth and ecological footprint from 1977 to 2013 in 11 newly industrialized countries. They concluded that there is an inverted U-shaped relationship. With the deepening of research, scholars have also discovered that there are N-type and inverted N-type relationships between some environmental indicators and economic growth [14,15,16,17,18]. Balin and Akan [19] used panel data methods to test the EKC hypothesis for 27 developed countries from 1997 to 2009 and found an N-shaped relationship between GDP per capita and CO_2_ per capita. Bandyopadhyay and Rej [20] examined the dynamic linkages between gross domestic product and CO_2_ emissions for India from 1978 to 2019, and the study confirmed the existence of an inverted N-type EKC.

### 2.2. Measurement of Environmental Quality in EKC Research

In the measurement of environmental quality, most scholars focus on a single environmental pollution indicator, starting from the three levels of industrial waste gas, wastewater, and solid waste [21,22,23]. For example, Sinha and Bhattacharya [24] estimated the EKC for 139 Indian cities, considering SO_2_ emissions. Adeel-Farooq et al. [25] analyzed the EKC hypothesis within the CH4 emission–economic growth nexus among the six ASEAN countries from 1985 to 2012. Lu et al. [26] focused on the relationship between GDP per capita and the volume of industrial wastewater discharge. Trujillo et al. [27] used solid waste data to prove the existence of the EKC in Colombia. However, it is difficult for a single indicator to express environmental quality, and the use of a single indicator when analyzing environmental quality often leads to biased results. Later, with further research, single indicators gradually transformed into comprehensive indicators, such as comprehensive indicators of environmental pollution gradually coming to be respected by the academic community. Manuel [28] used five air pollutants, such as carbon dioxide and sulfur dioxide, in Peru from 1980 to 2011 to form a comprehensive index of air pollution to test the EKC of the country and found that the country does not support the inverted U-shaped curve relationship.

### 2.3. EKC Research in China

Since 2000, many scholars have conducted research on the EKC in China. Tao et al. [29] empirically studied the relationship between environmental pollution and China’s economic growth using the country’s provincial data from 1985 to 2005. The study found an inverted U-shaped relationship between GDP and waste gas, wastewater, and solid waste. Zhou [30] used Chongqing’s economic and environmental statistics from 1995 to 2009 to analyze the relationship between industrial economic growth and the three industrial waste types and found an inverted N-shaped relationship between industrial per capita output and industrial wastewater. Jiang et al. [31] used Chinese provincial panel data from 1985 to 2005 to make an empirical estimate of the EKC model and found an inverted U-shaped relationship between per capita income and per capita emissions. Zhang and An [32] analyzed the dynamic relationship between economic growth and six specific industrial environmental pollution intensity indicators using data from Shanxi Province from 1995 to 2015 and found that three out of the six environmental pollution intensity responses were in the shape of an inverted U curve. The pollution intensity of COD represents an N-shape, while solid waste and waste gas show U-shaped curves. Riti et al. [33] applied different estimation techniques to study the relationship between China’s GDP per capita and CO_2_ emissions from 1970 to 2015, and the results supported the EKC hypothesis using different techniques. Fan and Zheng [34] used regression method to analyze the relationship between GDP per capita and emissions of industrial waste in Sichuan Province from 1985 to 2010 and suggested that there exists an inverted N-shaped or a U-shaped relationship between economic development and environmental pollution. Li et al. [35] examined the EKC relationship between economic growth and energy consumption in China with the provincial panel data from 2000 to 2017, and the results supported the inverted N-type EKC.

### 2.4. Research Gap

The existing literature has the following shortcomings: First, the current research on EKC in China focuses on the national and provincial levels. There is little research on the city level. China has a vast territory, and the environmental conditions of its various regions are significantly different. As a division unit, the city can better reflect the characteristics of adapting measures to local conditions. Second, when selecting environmental quality indicators, most studies only use a single indicator or multiple single indicators to measure the status of environmental quality, which cannot fully reflect the comprehensive changes in environmental quality and may lead to deviations in conclusions when analyzing problems. Finally, the selection of indicators has tended to be higher at the pollution level. Although some studies have constructed comprehensive indicators of environmental pollution, they do not consider the absorption and self-purification of the environment itself and cannot truly reflect the real situation of environmental quality.

Compared with previous studies, the contributions of this paper are threefold. First, we selected 74 key environmental monitoring cities in China from 2005 to 2017 as the research objects, conducted an empirical analysis of the changes in the EKC in China in recent years, and tried to determine the complex relationship between the environmental quality of China’s cities and economic growth and verify whether there is an EKC curve in China. Second, a comprehensive environmental quality index was established, and the weight of the index was objectively determined using the vertical and horizontal grading method, so that the index can represent the overall environmental quality to the greatest extent and make up for the dilemma of previous empirical research using a single or several independent environmental quality indicators not revealing the overall environmental quality. Third, a new comprehensive evaluation index system for urban environmental quality was constructed. Ten pollutants in the atmosphere, soil, and water were selected to reflect the environmental pollution status, and six environmental absorption indexes were selected to measure the environmental self-purification capacity, which can more accurately reflect the environmental quality status.

## 3. Research Design

### 3.1. Sample Selection and Data Source

The research scope covered all provincial administrative regions except Tibet, Taiwan, Hong Kong, and Macao, specifically the first batch of cities to implement the new air quality standards announced in 2012. The list of specific cities is shown in Appendix A. Among them, there are 19 first-tier and new first-tier cities, 28 second-tier cities, and 27 third-tier and below cities. The data for cities from 2005 to 2017 mainly came from statistical yearbooks and online databases. Among them, sulfur dioxide, smoke (dust) emissions, domestic garbage removal, chemical fertilizer use, industrial solid waste emissions, nitrogen oxide emissions, and ammonia nitrogen emissions came from the “China Statistical Yearbook”; energy consumption and electricity consumption data were provided by the “China Energy Statistical Yearbook” and the carbon dioxide emissions of each city were measured by the standard quantity conversion coefficient and the carbon emission coefficient; and the pesticide data were provided by the “China Rural Statistical Yearbook.” The green area, average relative humidity of major cities, wetland area, forest area, and annual precipitation data were provided by the “China Statistical Yearbook” and the data on total water resources came from the “China Water Resources Bulletin.” The rest of the data mainly came from the Wind database.

### 3.2. Model Specification

To analyze the impact of economic growth on environmental quality, we used the empirical model equations of Grossman and Krueger [6] for reference. The specific model is as follows:

(1) $$EQ_{it}=\alpha_{it}+\beta_{1}PGDP_{it}+\beta_{2}PGDP^{2}{}_{it}+\beta_{3}PGDP^{3}{}_{it}+\beta_{4}Edu_{it}+\beta_{5}Tech_{it}+\beta_{6}Urban_{it}+\beta_{7}Stru_{it}+\delta_{i}+\varepsilon_{it}$$

where *i* and *t* represent the city and year, respectively; *EQ* represents the urban environmental quality, which is measured by the logarithm of the comprehensive environmental quality index; *PGDP* represents the level of economic growth, measured by the logarithm of per capita GDP; Edu represents the level of education, which is measured by the logarithm of government investment in education; *Tech* refers to the technical level, which is measured by the logarithm of fiscal expenditure on science and technology; *Urban* refers to the urbanization rate, which is measured by the proportion of urban population in the permanent resident population; and *Stru* represents the industrial structure, which is measured by the proportion of the added value of the secondary industry in GDP.

### 3.3. Variable Descriptions

#### 3.3.1. Explained Variable: Urban–Environmental Quality

Since environmental quality is an abstract concept, it must be quantified when studying EKC. In contrast to previous studies, we constructed a comprehensive environmental quality index. This index is a composite of an environmental pollution index and an environmental absorption index.

The environmental pollution index was calculated from 10 environmental pollution indicators: the higher the pollution index, the more serious the pollution. The 10 environmental pollution indicators are listed in Table 1.

The environmental absorption index was calculated from six absorption indicators, which represent the absorption capacity of the pollutants in the area. This is a percentage concept and a positive index. The higher the value of the environmental absorption index, the stronger the self-purification ability. The six environmental absorption indicators are listed in Table 2.

The steps to calculate the comprehensive environmental quality index are as follows:

First, the linear proportion method is used to process the original index data dimensionless. Let $\left\{x_{ij}(t_{k})\right\}$ denote the value of the *j*-th index of sample *i* at time $t_{k}$, (*i* = 1, …, m; *j* = 1, …, *m*; *k* = 1, …, *m*). Therefore,

(2) $$x_{ij}^{*}(t_{k})=x_{ij}(t_{k})/m_{j}(t_{k})$$

where $x_{ij}^{*}(t_{k})$ is the normalized value, *i* is the city, *j* is the index, $x_{ij}(t_{k})$ is the original index value, and $m_{j}(t_{k})$ is the minimum value of the *j*-th index.

Secondly, calculate the real symmetric matrix $H_{k}=A_{k}^{T}A_{k}(k=1,2,\ldots ,N)$ and

(3) $$A_{k}=\left(\begin{array}{ccc} x_{11}^{}(t_{k}) & \ldots & x_{1m}^{}(t_{k})\\ \ldots & & \ldots \\ x_{n1}^{}(t_{k}) & \ldots & x_{nm}^{}(t_{k}) \end{array}\right)\;\;\;\;\;\;\;\;\;k=1,\ldots ,N$$

Third, the maximum eigenvalue of the real symmetric matrix H and the corresponding standard eigenvector $\lambda^{\prime }$.

$H=\sum_{k=1}^{N}H_{k}$ is a symmetric matrix of order m×m, *k* = 1, …, *N*.

Fourthly, normalize the standard feature vector $\lambda^{\prime }$ to determine the combined weight vector $\omega_{j}$.

Fifthly, calculate the absolute environmental pollution index and absolute environmental absorption index $P_{i}(t_{k})$.

(4) $$P_{i}(t_{k})=\sum_{j=1}^{n}\omega_{j}x^{*}{}_{ij}(t_{k}),\;\;k=1,\ldots ,N;\;\;i=1,\ldots ,m$$

$P_{i}(t_{k})$ is the index value of the *i*-th evaluation object in year $t_{k}$; $\omega_{j}$ is the weight value of the *j*-th indicator.

Sixth, the absolute total environmental quality index is calculated using Formula (5).

(5) $$EQ_{i}(t_{k})=A_{i}(t_{k})\times (1-B_{i}(t_{k}))$$

$EQ_{i}(t_{k})$ represents the absolute total environmental quality index of the *i*-th evaluation object in year $t_{k}$. $A_{i}(t_{k})$ represents the absolute environmental pollution index of the *i*-th evaluation object in year $t_{k}$. $B_{i}(t_{k})$ represents the absolute environmental absorption index of the *i*-th evaluation object in year $t_{k}$.

It can be seen that the comprehensive environmental quality index refers to the comprehensive evaluation index obtained by mathematical calculation of the pollution index and the absorption index. The larger the comprehensive index, the worse the environmental quality; the smaller the comprehensive index, the better the environmental quality. The calculation results of the comprehensive environmental quality index are presented in Appendix B.

#### 3.3.2. Explanatory Variables: Economic Growth

We used per capita GDP to measure economic growth because per capita GDP is more suitable for reflecting the impact of changes in actual income levels on environmental quality. Because the population size and natural endowment of China’s cities are quite different relative to the total GDP, the per capita GDP was more suitable for the statistical analysis of this study.

#### 3.3.3. Control Variables

This study also set up a series of control variables. Xie et al. [36] believe that technological progress has a profound impact on environmental quality, and technological progress can generally be divided into scientific and technological innovation, policy regulation, and scientific and technological investment. This study selected fiscal expenditure on science and technology to measure technological progress, which not only reflects the effect of government regulation, but also highlights the impact of scientific and technological investment. In addition, the level of urbanization, industrial structure, and education investment also have a nonnegligible impact on environmental quality [37,38,39]. Therefore, this study selected urbanization rate, industrial structure, and education level as control variables.

#### 3.3.4. Descriptive Statistics

The descriptive statistics of the main variables are shown in Table 3.

## 4. Empirical Results

### 4.1. Model Selection

This study used panel data. There are three forms of panel regression models: pooled regression models, fixed effect models, and random effect models. Among them, the F test is used to judge whether to choose the pooled regression model or the fixed effect model, and the Hausman test is used to judge whether to choose the fixed effect model or the random effect model. In this study, the F test was implemented first. The results showed that F (73, 807) = 104.86, prob > F = 0.0000. The p-value was less than 0.05, so the original assumption that the real model was a pooled regression model was rejected, and the fixed effect model was better than the mixed regression model. Then, the Hausmann test was carried out. The results showed that chi2(7) = 31.25, Prob > chi2 = 0.0001. Therefore, the original hypothesis that the random effect model was better was rejected at the 5% significance level. Finally, the fixed effect model was used for the estimation.

### 4.2. Regression Results

The regression results are shown in Table 4. For the total sample, $\beta_{1}$ = −70.7346, $\beta_{2}$ = 6.8570, and $\beta_{3}$ = −0.2212 met the condition $\beta_{1}$ < 0, $\beta_{2}$ > 0, $\beta_{3}$ < 0, and they were significant at the 1% level, which shows that the relationship between China’s urban–environmental quality and economic growth meets the inverted N-type Kuznets curve. According to the formulas $\exp\left[\beta_{2}+\sqrt{\beta_{2}^{2}-3\beta_{1}\beta_{3}}/\left(3\beta_{3}\right)\right]$ and $\exp\left[\beta_{2}-\sqrt{\beta_{2}^{2}-3\beta_{1}\beta_{3}}/\left(3\beta_{3}\right)\right]$, we calculated that the two inflection points are CNY 25,667.99 and CNY 52,680.47, respectively. The EKC of the total sample can be divided into three stages: the first stage is when the per capita GDP of the total sample is less than CNY 25,667.99. At this time, environmental quality will continue to improve with economic growth. This stage is mainly due to the gradual start of industrialization, the gradual establishment of the industrial system, and the gradual elimination of the original and backward modes of production. The second stage is when the per capita GDP of the total sample is between CNY 25,667.99 and 52,680.47. At this time, the environmental quality will continue to deteriorate with economic growth. This is because with the further development of industrialization, energy consumption increases, nonintensive industries create a lot of sewage, the gradual expansion of urbanization brings heavy domestic pollution pressure, science and technology are not developed, and the lack of sufficient pollution treatment capacity leads to the continuous deterioration of the environment. The third stage is when the per capita GDP of the total sample is greater than CNY 52,680.47. At this time, environmental quality will continue to improve with economic growth. This is because with the continuous promotion of industrialization, the industry is gradually transformed and upgraded to intensive and intelligent, the intensity of energy conservation and emissions reduction is enhanced, and the development of green science and technology greatly enhances the pollution absorption capacity; therefore, the environmental quality gradually improves. By 2017, the average per capita GDP of 74 key environmental monitoring cities in the whole sample was CNY 73,261, which was greater than the second inflection point value of CNY 52,680.47. Therefore, in fact, the sample cities have been in the third stage of the inverted N-shaped curve; that is, with the continuous improvement of per capita GDP, the environmental quality will continue to improve, which is basically consistent with the current reality of China.

For the first-tier and new first-tier cities, $\beta_{1}$ = −122.6499, $\beta_{2}$ = 11.7356, and $\beta_{3}$ = −0.3740 met the condition $\beta_{1}$ < 0, $\beta_{2}$ > 0, $\beta_{3}$ < 0, and they were significant at the 1% level, which shows that the relationship between environmental quality and economic growth in China’s first-tier and new first-tier cities meets the inverted N-type Kuznets curve. According to the formulas $\exp\left[\beta_{2}+\sqrt{\beta_{2}^{2}-3\beta_{1}\beta_{3}}/\left(3\beta_{3}\right)\right]$ and $\exp\left[\beta_{2}-\sqrt{\beta_{2}^{2}-3\beta_{1}\beta_{3}}/\left(3\beta_{3}\right)\right]$, we calculated that the two inflection points are CNY 20,657.43 and CNY 50,891.33, respectively. The EKC of first-tier and new first-tier cities can be divided into three stages: the first stage is when the per capita GDP of first-tier and new first-tier cities is less than CNY 20,657.43, and the environmental quality will continue to improve with economic development. This stage is mainly due to the first-tier and new first-tier cities taking the lead in reform and opening up, starting industrial development, and changing the backward mode of production. In the second stage, when the per capita GDP of first-tier and new first-tier cities is between CNY 20,657.43 and CNY 50,891.33, the environmental quality will deteriorate with economic growth. This is because most first-tier and new first-tier cities have weak absorption capacities. With the further development of industrialization and the intensification of energy consumption, the environmental impact will be amplified. Moreover, the scale of such cities is huge, and the rapid expansion of urbanization also brings heavy domestic pollution pressure, so the environmental quality will continue to deteriorate. The third stage is when the per capita GDP of the first-tier cities and new first-tier cities is greater than CNY 50,891.33. At this time, environmental quality will continue to improve with economic growth. This is because, with continuous economic growth, the first-tier cities and new first-tier cities have a good industrial foundation, the industrial transformation is relatively smooth, the intelligent and science and technology-oriented industries have been gradually formed, the pollution emissions are lower, higher education in these cities is developed, and the industry–university–research system is sound, so it can save energy and reduce emissions through green science and technology. At the same time, this kind of urban population has a high level of education, strong awareness of environmental protection and responsibility, and can reduce domestic pollution to a certain extent. Therefore, the overall environmental quality will continue to improve. As of 2017, the per capita GDP of the first-tier and new first-tier cities reached CNY 106,070.2, which is greater than the second inflection point value of CNY 50,891.33. Therefore, the first-tier and new first-tier cities have been in the third stage of the inverted N-shaped curve; that is, with the continuous improvement of per capita GDP, the environmental quality will gradually improve. At the same time, it can also be found that the inflection point value of the first-tier and new first-tier cities is significantly less than that of the total sample, which means that the first-tier and new first-tier cities can pass the inflection point earlier, which is inseparable from the strong government environmental regulation measures, good economic foundation, developed level of education and science and technology, and people’s strong sense of environmental protection awareness and responsibility.

For the second-tier cities, $\beta_{1}$ = −117.4796, $\beta_{2}$ = 11.1052, and $\beta_{3}$ = −0.3495, meeting the condition $\beta_{1}$ < 0, $\beta_{2}$ > 0, $\beta_{3}$ < 0, and the results were significant at the 1% level, which shows that the relationship between environmental quality and economic growth in China’s second-tier cities meets the inverted N-type Kuznets curve. According to the formulas $\exp\left[\beta_{2}+\sqrt{\beta_{2}^{2}-3\beta_{1}\beta_{3}}/\left(3\beta_{3}\right)\right]$ and $\exp\left[\beta_{2}-\sqrt{\beta_{2}^{2}-3\beta_{1}\beta_{3}}/\left(3\beta_{3}\right)\right]$, we calculated that the two inflection points are CNY 23,391.74 and CNY 50,161.35, respectively. The EKC of second-tier cities can be divided into three stages: the first stage is when the per capita GDP of second-tier cities is less than CNY 23,391.74. At this time, environmental quality will continue to improve with economic growth. This stage is mainly due to the gradual industrialization process of second-tier cities under the promotion of reform and opening up, which is realized through changes in the economic system and industrial structure. In the second stage, when the per capita GDP of the second-tier cities is between CNY 23,391.74 and CNY 50,161.35, the environmental quality will deteriorate with economic growth. This is because most of the second-tier cities are dominated by secondary industries, and the added value of these industries in some cities accounts for more than 60%. The large industrial scale will inevitably lead to an increase in energy consumption, the nonintensive and extensive industrial model also intensifies the emission of pollution, and there is an obvious gap between the scientific research strength and the scientific and technological level of such cities compared with the first-tier and new first-tier cities, so they cannot achieve emission reductions well, resulting in the continuous deterioration of environmental quality. The third stage is when the per capita GDP of the second-tier cities is greater than CNY 50,161.35. At this time, environmental quality will continue to improve with economic growth. This is because, with continuous economic growth, second-tier cities continue to realize industrial transfer, and some high-energy-consumption and high-pollution industries are transferred to the third-tier and below cities, so that the industrial structure can be upgraded. At the same time, through the introduction of talent and technology, energy conservation and emissions reduction abilities have also been significantly enhanced, thus improving the environmental quality. As of 2017, the per capita GDP of second-tier cities was CNY 83,722, exceeding the second inflection point value of CNY 50,161.35. Therefore, at present, second-tier cities have also been in the third stage of the inverted N-shaped curve; that is, with the continuous improvement of per capita GDP, the environmental quality will gradually improve.

For the third-tier and below cities, $\beta_{1}$ = 7.5731, $\beta_{2}$ = −0.6571, and $\beta_{3}$ = 0.0188, meeting the condition $\beta_{1}$ > 0, $\beta_{2}$ < 0, $\beta_{3}$ > 0, but the results were not significant. Therefore, the EKC is not established in China’s third-tier and below cities. In terms of the control variables, except for industrial structure and fiscal expenditure on science and technology, the other indicators were not significant. In 2017, most of the third-tier and below cities were smaller, coastal, prefecture-level cities and provincial capitals of remote inland provinces, which were not developed as a whole. In 2017, the average per capita GDP of third-tier and below cities was only CNY 56,705, less than half of that of first-tier and new first-tier cities. This is because these cities are at a stage of industrialized development. In 2017, the average proportion of secondary industries in the third-tier and below cities was only 42.38%, especially in cities such as Hohhot and Urumqi; the industrial structure was relatively backward; they were still dominated by traditional agriculture, tourism, and other industries; and the pressure brought by economic activities on the environment was not heavy. Moreover, in terms of education investment, the average investment amount of third-tier and below cities in 2017 was only half of that of second-tier cities, less than one-tenth of the average level of first-tier and new first-tier cities, and the total amount was small. At the same time, the population scale of these cities is small, and their attraction to the foreign population is weak. Moreover, cities such as Lishui have a strong absorption capacity for the environment itself, and the expansion of urbanization has little impact on environmental quality. Therefore, the impact of economic growth on environmental quality is not obvious, so the EKC of the third-tier and below cities is not significant.

### 4.3. Robustness Check

First, considering that panel data may have heteroscedasticity, sequence correlation, and other problems, this study adopted a panel-corrected standard error (PCSE) for the robustness test. PCSE can introduce the residual term into the diagonal matrix on the basis of preserving ordinary least squares to estimate the model more accurately. The regression results of the robustness test are presented in Table 5. It can be seen that the basic conclusion has not changed, so the regression result estimated in this study is robust.

**Figure 2:**
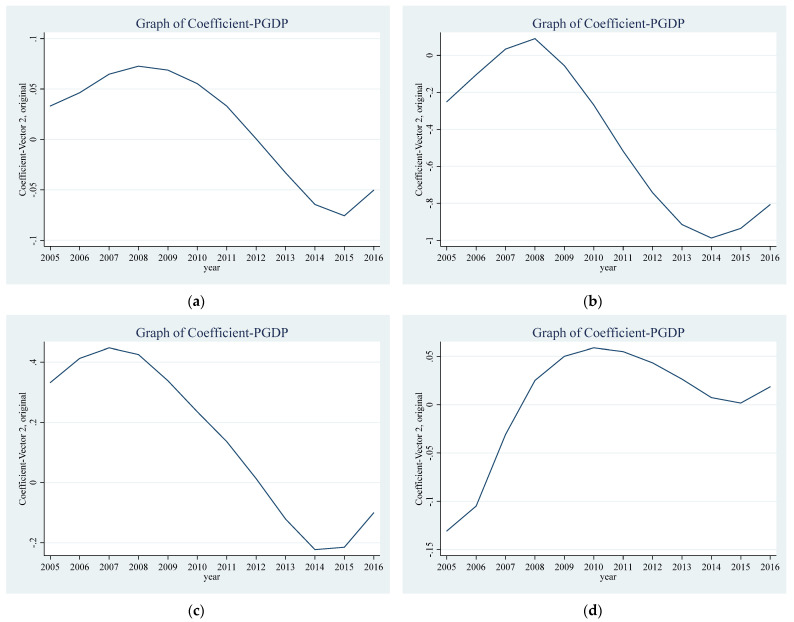
Time-varying responsiveness of EQ to changes in PGDP. (**a**) represents the total sample, (**b**) represents the first-tier and new first-tier cities, (**c**) represents the second-tier cities, (**d**) represents the third-tier and below cities.

Second, parametric regression models may cause model misspecification [40]. To solve this problem, this study drew on the practice of Sadik-Zada and Loewenstein [41] and Li et al. [42], employing the nonparametric time-varying coefficient panel data model with fixed effects. This method does not impose a priori a specific functional form for the relationship between the variables of interest, which enables the data to “speak for themselves” [43]. The results of the nonparametric estimation are shown in Figure 2. Figure 2A–D show the estimation results for the total sample, first-tier and new first-tier cities, second-tier cities, and third-tier and below cities, respectively. It can be found that the curves of the total sample, first-tier and new first-tier cities, and second-tier cities are all N-shaped. The estimated coefficient of PGDP is shown here. Figure 2A–C show that the coefficient of PGDP gradually becomes negative over time; that is, the environmental quality gradually improves with economic growth and conforms to the EKC curve.

## 5. Discussion

Many researchers have used different data sources and different research methods to empirically test the existence of the EKC. Due to the lack of comprehensive environmental indicators, environmental pollution indicators are divided into three categories (air pollution, water pollution, and solid waste). Research shows that although the environmental transformation path in the process of economic growth is not invariable, there is a clear relationship between some environmental indicators and income levels for specific pollutants. However, not every environmental indicator conforms to the EKC’s inverted U model. Some studies have found that there may be N-type and inverted N-type relationships between environmental indicators and the economy, which vary according to different regions and environmental indicators. The findings of this study are consistent with those of Bandyopadhyay and Rej [20], Fan and Zheng [34], and Li et al. [35]. These studies confirmed the existence of an inverted N-type EKC. This shows that with the development of China’s economy, the country’s urban–environmental quality is on a trend of first rising, then falling, and then rising. Through the calculation of the turning point, whether it is the full sample, first-tier and new first-tier cities, or second-tier cities, the second turning point has now been passed, and the environmental quality will continue to improve with economic growth.

## 6. Conclusions

Based on the EKC as the main theoretical basis, this study selected 74 key environmental monitoring cities in China as the research object, constructed a comprehensive index of environmental quality, made a detailed study and demonstration of whether the EKC hypothesis of China’s urban environment is tenable, and determined the inflection point position. It was found that the EKC based on the comprehensive index of environmental quality showed an inverted N-shape in the total sample, first-tier and new first-tier cities, and second-tier cities, while it was not significant in the third-tier cities, which shows that with the economic growth of the total sample, first-tier and new first-tier cities, and second-tier cities, the environmental quality first rose, then declined, and then rose. At the same time, through the calculation of the inflection point, at present, the total sample, first-tier cities and new first-tier cities, and second-tier cities can be said to have passed the second inflection point, and the environmental quality will continue to improve with economic growth. This study makes important theoretical and practical contributions to the literature. On the one hand, this paper calculated the comprehensive index of environmental quality from the perspective of pollution and absorption for the first time, better measured the real local environmental situation, compensated for the deficiency of previous EKC studies only focusing on environmental pollution indicators, and further enriched the EKC theory. On the other hand, the research conclusion of this paper showed that China can cross the inflection point of the EKC under the condition of a low-income level. Therefore, by vigorously developing cleaner production technologies and enhancing residents’ awareness of environmental protection, it is entirely possible to achieve an improvement in environmental quality at a lower income level than expected, which provides a useful reference for other developing countries. However, due to the influence of the data collection, this study has some limitations. For example, the sample period selected in this paper was 2005–2017. In a follow-up study, it will be necessary to constantly update the data and conduct a more in-depth analysis of the changes and characteristics of EKC.

## Figures and Tables

**Table 1:** Environmental pollution indicators.

Sub-Index	Pollution Receptor	Sub-Indicator	Index Unit	Time Interval	Indicator Attributes
Environmental pollution	Atmosphere	Total nitrogen oxide emissions	Ten thousand tons	2005–2017	Negative
		Total sulfur dioxide emissions	Ten thousand tons	2005–2017	Negative
		Total smoke (dust) emissions	Ten thousand tons	2005–2017	Negative
		Carbon dioxide emissions	Billion tons	2005–2017	Negative
	Soil	Solid waste generation	Ten thousand tons	2005–2017	Negative
		Domestic garbage removal volume	Ten thousand tons	2005–2017	Negative
		Chemical fertilizer application rate	Ten thousand tons	2005–2017	Negative
		Pesticide usage	Ton	2005–2017	Negative
	Water body	Chemical oxygen demand	Ten thousand tons	2005–2017	Negative
		Ammonia nitrogen emissions	Ten thousand tons	2005–2017	Negative

**Table 2:** Environmental absorption indicators.

8	Purify Receptor	Sub-Indicator	Index Unit	Time Interval	Indicator Attributes
Environmental absorption	Atmosphere, Soil, Water body	Urban green area	Hectares	2005–2017	Positive
		Average relative humidity of major cities	Percentage	2005–2017	Positive
		Annual precipitation	Millimeter	2005–2017	Positive
		Total water resources	One hundred million cubic meters	2005–2017	Positive
		Wetland area	Thousand hectares	2005–2017	Positive
		Forest area	Ten thousand hectares	2005–2017	Positive

**Table 3:** Descriptive statistics.

Variable	Obs	Mean	St.d.	Min	Max
EQ	962	4.2916	0.6400	3.0330	5.5500
PGDP	962	10.7412	0.5470	9.6640	11.5927
Edu	962	3.9330	0.8603	2.2965	5.4603
Tech	962	1.8098	1.1874	−0.2689	4.0972
Urban	962	59.4090	15.6099	34.5600	88.7000
Stru	962	48.0312	7.1854	31.8000	58.8000

Note: EQ represents the urban environmental quality, PGDP represents the level of economic growth, Edu represents the level of education, Tech refers to the technical level, Urban refers to the urbanization rate, Stru represents the industrial structure.

**Table 4:** Regression results.

Variable	Total Sample	First-Tier and New First-Tier Cities	Second-Tier Cities	Third-Tier and Below Cities
PGDP	−70.7346 ***	−122.6499 ***	−117.4796 ***	7.5731
	(−5.07)	(−4.16)	(−4.12)	(0.34)
PGDP2	6.8570 ***	11.7356 ***	11.1052 ***	−0.6571
	(5.22)	(4.27)	(4.17)	(−0.31)
PGDP3	−0.2212 ***	−0.3740 ***	−0.3495 ***	0.0188
	(−5.37)	(−4.38)	(−4.21)	(0.28)
Edu	−0.0617 **	−0.1929 ***	−0.0219	−0.0130
	(−2.48)	(−3.58)	(−0.47)	(−0.36)
Tech	0.0512 ***	0.1191 ***	0.0409 *	0.0359 *
	(3.47)	(4.09)	(1.74)	(1.88)
Urban	0.0043 ***	0.0048 **	0.0055 **	0.0034
	(2.91)	(2.07)	(2.29)	(1.25)
Stru	0.0163 ***	0.0067	0.0327 ***	0.0150 ***
	(7.01)	(1.65)	(7.42)	(4.40)
Cons	246.2057 ***	431.6935 ***	416.0340 ***	−25.6340
	(4.99)	(4.11)	(4.11)	(−0.33)
City effect	Yes	Yes	Yes	Yes
F-statistic	36.45	29.92	16.40	7.03
Within_R2	0.2402	0.5091	0.2761	0.1452
N	962	247	364	351

Note: ***, **, and * represent significance at the 1%, 5%, and 10% levels, respectively, and the values in parentheses are t statistics.

**Table 5:** Robustness check.

Variable	Total Sample	First-Tier and New First-Tier Cities	Second-Tier Cities	Third-Tier and Below Cities
PGDP	−61.3897 ***	−123.7944 ***	−96.4978 ***	−5.5735
	(−4.39)	(−4.59)	(−2.83)	(−0.32)
PGDP2	5.9490 ***	11.8347 ***	9.1519 ***	−0.4767
	(4.52)	(4.69)	(2.87)	(−0.29)
PGDP3	−0.1918 ***	−0.3769 ***	−0.2889 ***	0.0135
	(−4.64)	(−4.80)	(−2.92)	(0.26)
Edu	−0.0471 *	−0.0893 *	−0.0057	−0.0135
	(−1.69)	(−1.82)	(−0.18)	(−0.33)
Tech	0.0369 **	0.0596 ***	0.0192 *	0.0489 *
	(2.03)	(2.71)	(1.62)	(1.71)
Urban	0.0031 *	0.0033 *	0.0052 *	0.0002
	(1.69)	(1.75)	(1.97)	(0.05)
Stru	0.0153 ***	0.0085	0.0265 ***	0.0132 ***
	(4.71)	(1.64)	(5.83)	(2.84)
Cons	215.6289 ***	436.6523 ***	340.7910 ***	−19.0294
	(4.37)	(4.54)	(2.80)	(−0.31)
City effect	Yes	Yes	Yes	Yes
Wald-statistic	5256.74	74,703.11	4682.38	16,097.97
R2	0.9444	0.9579	0.9258	0.9446
N	962	247	364	351

Note: ***, **, and * represent significance at the 1%, 5%, and 10% levels, respectively, and the values in parentheses are t statistics.

## Data Availability

The data presented in this study are available on request from the corresponding author. The data are not publicly available due to privacy or ethical restrictions.

## Appendix A

**Table A1:** Seventy-four key environmental monitoring cities.

Level	City
First-tier and new first-tier cities	Beijing, Shanghai, Tianjin, Chongqing, Chengdu, Hangzhou, Chongqing, Wuhan, Xi’an, Suzhou, Tianjin, Nanjing, Changsha, Zhengzhou, Dongguan, Qingdao, Shenyang, Ningbo, Kunming (19).
Second-tier cities	Wuxi, Foshan, Hefei, Dalian, Fuzhou, Xiamen, Harbin, Jinan, Wenzhou, Nanning, Changchun, Shijiazhuang, Guiyang, Nanchang, Jinhua, Changzhou, Nantong, Jiaxing, Taiyuan, Xuzhou, Huizhou, Zhuhai, Zhongshan, Taizhou (Zhejiang), Lanzhou, Shaoxing, Haikou, Yangzhou (28).
Third-tier and below cities	Qinhuangdao, Handan, Xingtai, Baoding, Zhangjiakou, Chengde, Cangzhou, Langfang, Hengshui, Tangshan, Hohhot, Lianyungang, Huai’an, Yancheng, Zhenjiang, Taizhou (Jiangsu), Suqian, Huzhou, Quzhou City, Lishui, Zhoushan, Zhaoqing, Jiangmen, Lhasa, Xining, Yinchuan, Urumqi (27).

## Appendix B

**Table A2:** Comprehensive environmental quality index.

**City**	**2005**	**2006**	**2007**	**2008**	**2009**	**2010**	**2011**	**2012**	**2013**	**2014**	**2015**	**2016**	**2017**	**Mean**	**Rank**
Lhasa	1.55	1.62	1.41	0.89	0.83	0.37	5.15	5.66	5.46	5.7	6.93	7.12	2.31	3.57	1
Haikou	1.52	1.53	10.56	10.82	12.28	13.45	0.85	0.88	0.89	0.93	1.13	1.37	4.81	4.68	2
Zhoushan	20.76	19.63	22.61	20.53	21.92	22.97	21.28	19.64	19.82	18.2	16.68	19.34	2.23	20.28	3
Lishui	21.08	21.04	21.71	19.71	21.99	24.98	29.55	25.42	23.98	23.97	25.47	23.37	5.65	23.52	4
Xiamen	46.22	46.58	34.33	36.17	35.13	44.58	19.35	16.3	15.57	12.88	14.45	14.5	8.5	28	5
Shenzhen	37.01	36.84	57.47	44.95	38.74	27.98	27.37	24.36	15.61	13.36	10.23	13.35	107.14	28.94	6
Zhaoqing	23.23	20.42	27.98	30.48	28.86	31.46	31.86	33.45	31.92	31.44	29.18	28.75	8.12	29.09	7
Quzhou	29.26	28.5	28.43	28.52	31.18	28.51	35.37	31.24	31.13	36.07	32.86	31.9	6.72	31.06	8
Nanchang	45.26	46.29	46	44.85	38.67	52.71	31.27	32	27.92	25.82	21.89	20.67	10.79	36.11	9
Suqian	29.65	29.05	28.55	34.04	32.69	37.32	41.59	43.85	41.56	42.9	36.94	36.73	17.35	36.24	10
Huizhou	31.17	31.84	39.46	45.1	42.23	42.92	44.77	46.28	43.44	41.27	36.77	36.85	11.4	40.18	11
Nanning	48.43	47.35	47.47	47.66	48.29	51.31	32.02	37.63	39.02	39.82	30.86	30.45	8.27	41.69	12
Jiangmen	39.35	38.27	49.27	50.09	44.53	44.16	48.32	46.97	43.04	39.15	34.75	35.04	9.46	42.75	13
Zhuhai	27.16	24.83	63.23	71.53	66.35	71.61	48.08	44.72	36.8	37.93	27.37	26.65	4.47	45.53	14
Zhongshan	42.94	41.8	49.7	54.43	50.54	51.8	46.02	48.42	43.87	41.31	39.19	37.38	5.27	45.62	15
Lianyungang	43.32	44.66	46.36	48.23	47.93	61.11	42.58	43.04	42.03	44.84	42.88	42.47	4.23	45.77	16
Huaian	43.88	40.5	44.88	43.6	47.9	47.96	50.29	56.97	56.03	56.43	50.97	50.65	4.69	49.17	17
Wenzhou	71.8	69.83	67.51	66.27	65.08	66.19	48.19	43.19	37.16	38.41	35.99	35.22	20.76	53.74	18
Jinhua	50.74	52.93	57.37	53.29	57.15	57.85	62.11	55.71	53.57	52.27	46.21	47.85	8.18	53.92	19
Changsha	68.83	65.34	70.93	75.64	70.62	76.54	43.88	44.42	38.91	37.51	34.4	34.57	13.96	55.14	20
Hefei	31.51	32.92	28.05	26.59	29.41	52.4	93.53	91.24	80.56	76.27	67.19	62.69	11.71	56.03	21
Xining	51.14	50.94	54.33	56.58	55.31	66.59	62.97	64.11	65.62	63.03	54.64	51.91	6.64	58.15	22
Shaoxing	74.01	73.84	70.87	59.82	61.68	54.51	51.11	53.21	50.76	52.49	49.73	48.99	12.8	58.42	23
Yinchuan	52.33	51.93	54.83	20.94	30.75	35.22	74.55	90.8	91.15	74.3	69.96	70.14	6.29	59.75	24
Kunming	49.54	50.01	56.33	50.89	49.38	52.12	77.77	75.62	81.22	65.79	60.35	60.15	5.96	60.68	25
Baoding	63.09	64.12	73.31	60.75	55.6	63.75	77.88	88.5	55.81	47.82	40.99	41.21	17.32	61.08	26
Changzhou	52.28	54.32	64.62	54.64	56.29	45.81	77.88	71.89	67.95	63.81	62.87	62.86	3.84	61.27	27
Taizhou (Zhejiang)	69.35	69.9	78.3	63.1	62.02	63.84	69.86	59.79	57.42	53.91	44.95	45.1	12.51	61.46	28
Taizhou (Jiangsu)	52.24	51.14	54.3	60.77	63.76	64.73	68.06	72.58	65.2	67.9	58.9	58.49	12.21	61.51	29
Hengshui	78.11	69.75	62.66	54.24	50.84	53.39	67.24	66.69	63.34	61.27	55.5	55.74	7.3	61.56	30
Xi’an	69.56	69.72	71.28	74.21	74.74	72.44	69.02	63.54	54	51.1	40.48	38.53	5.61	62.38	31
Nantong	63.1	61.53	59.39	61.65	70.46	76.62	70.2	70.14	60.1	60.76	54.2	54	26.07	63.51	32
Yangzhou	78.11	76.55	75.58	71.59	70.5	59.08	70.16	62.93	59.56	52.2	46.86	45.9	4.44	64.09	33
Yancheng	58.64	58.97	59.58	55.32	59.95	63.38	66.26	75.23	75.09	74.3	64.23	63.51	4.52	64.54	34
Zhenjiang	65.52	64.22	64.82	58.69	60.12	62.58	74.34	76.24	71.1	69.82	55.55	53.66	2.78	64.72	35
Huzhou	72.89	72.9	87.47	78	95.61	93.62	55.12	51.62	45.77	46.79	44.62	44.04	7.69	65.7	36
Chengdu	84.01	79.33	77.1	91.95	88.19	60.52	60.64	62.04	52.7	53.47	41.91	43.09	14.79	66.26	37
Guiyang	112.62	87.06	68.33	70.71	72.87	74.43	59.13	51.23	54.57	53.52	51.32	53.21	7.59	67.42	38
Lanzhou	51.65	57.4	58.32	58.19	63.59	71.65	92.36	87.57	85.37	77.46	64.46	58.51	3.79	68.88	39
Qinhuangdao	53.98	55.99	60.46	56.99	59.41	74.66	91.22	123.99	83.82	84.97	61.54	52.82	8.05	71.66	40
Jiaxing	73.01	79.21	84.04	79.38	82.46	75.16	80.24	72.66	69.42	70.53	59.56	59.22	11.17	73.75	41
Fuzhou	65.05	65.45	66.77	62.24	71.75	112.15	101.29	88.69	81.03	78.23	70.17	67.78	17.29	77.55	42
Changchun	61.23	61.11	65.25	65.16	71.62	126.65	94.68	92.99	89.65	90.46	83.31	95.17	8.33	83.11	43
Chengde	76.27	71.72	84.45	84.51	74.03	79.12	106.8	112.87	96.81	86.33	70.12	69.12	3.75	84.34	44
Langfang	76.83	71.97	76.84	68.84	65.33	68.85	102.24	102.61	101.83	104.12	94.17	95.1	1.92	85.73	45
Shenyang	53.46	61.7	87.15	88.35	101.86	104.7	93	97.53	107.95	105.9	87.95	93.49	19.51	90.24	46
Hangzhou	110.61	109.31	110.88	99.2	106.3	113.97	90.94	83.07	77.29	75.89	66.4	62.94	14.09	92.23	47
Jinan	82.77	82.7	86.85	93.15	95.04	96.52	110.23	105.67	94.43	90.72	88.52	89.04	8.76	92.97	48
Qingdao	113.85	109.96	102.89	101.89	105.35	96.34	93.85	94.76	83.42	78.47	79.61	79.53	5.7	94.99	49
Harbin	71.18	73.92	82.46	94	94.21	94.58	113.69	149.58	104.58	108.3	94.63	100.65	22.25	98.48	50
Guangzhou	151.85	145.35	161.19	179.29	94.73	90.73	76.75	64.91	63.97	59.08	48.7	49.36	32.09	98.82	51
Zhangjiakou	105.29	100.66	107.68	105.51	99.09	97.12	113.93	109.93	104.4	103.33	90.08	88.99	3.29	102.17	52
Urumqi	94.02	100.05	105.86	106.02	99.91	125.58	125.61	129.1	112.15	96.8	74.37	70.91	9.25	103.37	53
Dalian	122.44	123.37	89.9	99.43	102.42	100.19	120.55	124.43	105.21	100.38	92.09	95.66	19.58	106.34	54
Dongguan	125.41	113.84	125.26	128.36	107.46	107.57	117.43	112.44	105.4	98.53	84.48	82.97	10.66	109.09	55
Xingtai	103.69	106.38	106.95	95.58	97.91	102.35	135.38	133.94	134.31	145.32	104.43	111.08	4.41	114.75	56
Hohhot	125.19	127.63	116.13	100.29	95.19	117.18	162.04	149	125.84	112.09	94.68	87.25	8.28	117.71	57
Cangzhou	100.97	107.65	111.82	86.41	86.42	102.25	158.54	145.47	141.41	135.3	118.54	129.18	3.72	118.67	58
Foshan	128.62	123.07	145.09	150.25	132.57	133.16	126.19	126.13	111.84	105.92	95.14	93.09	13.42	122.59	59
Taiyuan	120.78	114.34	112.61	115.01	107.56	127.18	154.66	154.57	129.05	123.98	110.42	107.98	8.46	123.18	60
Xuzhou	128.69	111.28	103.07	117.56	108.54	163.88	168.5	179.2	157.89	139.74	108.47	107.6	4.56	132.87	61
Zhengzhou	141.88	137.46	167.24	138.45	129.92	144.59	157.92	153.89	138.39	122.17	107.35	107.51	15	137.23	62
Wuhan	178.53	177.38	169.93	170.1	164.8	154.16	127.83	120.83	109.11	104.37	93.97	92.78	24.15	138.65	63
Nanjing	153.69	150.93	146.2	148.51	146.91	158.55	157.8	136.56	128.88	125.73	114.28	109.34	20.16	139.78	64
Wuxi	195.72	192.59	221.23	179.36	177.87	161.18	137.59	125.25	115.39	109.47	100.67	98.87	10.05	151.27	65
Beijing	196.63	209.92	219.65	164.4	166.57	192.84	163.86	155.96	146.39	134.16	118.97	80.67	14.92	162.5	66
Shijiazhuang	107.52	192.77	188.69	148.37	143.2	176.35	217.52	201.6	203.74	174.31	141.39	134.31	19.4	169.15	67
Ningbo	151.51	153.53	184.31	159.48	188.66	222.85	223.75	207.99	191.21	154.22	130.07	128.52	16.48	174.68	68
Handan	151.64	151.45	274.32	164.2	167.72	177.95	239.98	224.91	216.96	198.37	170.2	156.05	7.12	191.14	69
Tianjin	195.82	194.98	218.78	213.37	226.24	249.15	320.09	303.95	287.56	266.69	233.56	134.87	29.64	237.09	70
Suzhou	288.83	279.68	298.62	289.84	257.24	332.67	234.92	217.72	198.47	191.93	168.78	169.81	9.58	244.04	71
Shanghai	482	478.94	474.55	463.59	407.09	422.84	380.73	352.91	333.67	297.81	270.78	155.59	17.68	376.72	72
Tangshan	274.47	340.17	397.96	397.05	392.54	406.84	458.21	447.29	425.59	401.84	377.26	360.89	33.48	390.01	73
Chongqing	377.84	419.74	423.32	394.8	395.73	430.59	457.02	436.96	418.92	409.58	377.12	252.34	65.03	399.51	74
